# The characteristics of protein-glutaminase from an isolated *Chryseobacterium cucumeris* strain and its deamidation application

**DOI:** 10.3389/fmicb.2022.969445

**Published:** 2022-08-09

**Authors:** Ruidan Qu, Tian Dai, Jiajing Wu, Aitian Tian, Yanfang Zhang, Li Kang, Wei Ouyang, Congli Jin, Jinjin Niu, Zhen Li, Zhongyi Chang, Deming Jiang, Jing Huang, Hongliang Gao

**Affiliations:** ^1^School of Life Sciences, East China Normal University, Shanghai, China; ^2^School of Health & Social Care, Shanghai Urban Construction Vocational College, Shanghai, China

**Keywords:** protein deamidation enzyme, *Chryseobacterium cucumeris*, purification, identification, soy protein isolate

## Abstract

Protein-glutaminase (PG), a deamidation enzyme commercially derived from *Chryseobacterium proteolyticum*, is used to improve the solubility and other functional properties of food proteins. In this study, a new PG-producing strain, *Chryseobacterium cucumeris* ZYF120413-7, was isolated from soil, and it had a high PG yield and a short culture time. It gave the maximum PG activity with 0.557 U/ml on Cbz-Gln-Gly after 12 h of culture, indicating that it was more suitable for PG production. The enzyme activity recovery and purification fold were 32.95% and 161.95-fold, respectively, with a specific activity of 27.37 U/mg. The PG was a pre-pro-protein with a 16 amino acids putative signal peptide, a pro-PG of 118 amino acids, and a mature PG of 185 amino acids. The amino acid sequence identity of PG from strain ZYF120413-7 was 74 and 45%, respectively, to that of PG from *C. proteolyticum* 9670^T^ and BH-PG. The optimum reaction pH and temperature of PG was 6 and 60°C, respectively. Enzyme activity was inhibited by Cu^2+^. The optimum PG substrate was Cbz-Gln-Gly, and the *K_m_* and *V_max_* values were 1.68 mM and 1.41 μM mg protein^−1^ min^−1^, respectively. Degree of deamidation (DD) of soy protein isolate (SPI) treated by purified PG was 40.75% within the first 2 h and 52.35% after 18 h. These results demonstrated that the PG from *C. cucumeris* ZYF120413-7 was a promising protein-deamidating enzyme for improving the functionality of food proteins.

## Introduction

In recent years, plant-based proteins have surpassed animal proteins due to their low cost, energy efficiency, and environmental friendliness (Liu et al., 2022). Some plant-based proteins have been used to replace meat (Schreuders et al., 2019), milk (Lipan et al., 2020), and other dairy products (Wilbanks, 2017). However, the application of plant-based proteins in the food industry is limited due to their poor functional properties, such as water solubility, emulsifying properties, foaming ability, and gelling ability. One of the most efficient methods for improving the functional properties of food-based proteins is deamidation. Protein deamidation is a reaction that converts amide groups in proteins into acid residues (carboxyl groups) *via* the liberation of ammonia (Liu et al., 2022). As the number of negatively charged carboxyl groups increases, the isoelectric point of proteins decreases, and protein solubility improves in many mildly acidic food systems (Chen et al., 2021). Deamidation can be classified as chemical deamidation and enzymatic deamidation. Enzymatic deamidation has several advantages over chemical deamidation, including high specificity, mild reaction conditions, and food safety (Suppavorasatit et al., 2011; Liu et al., 2022). Enzymes that have been used for protein deamidation include transglutaminase (Ohtsuka et al., 2001), glutaminase (Fang et al., 2020), and peptidoglutaminase (Hamada, 1991). However, these enzymes are not suitable for protein deamidation because the primary catalytic reactions of transglutaminases are not deamidation, and primary substrates of glutaminase and peptidoglutaminases are not proteins.

A protein deamidation enzyme, named protein-glutaminase (PG; EC 3.5.1.44), was discovered in the culture supernatant of *Chryseobacterium protoelyticum* strain 9670^T^ isolated from rice field soil in 2000 (Yamaguchi and Yokoe, 2000). Because *C. protoelyticum* was shown to be non-pathogenic, and PG was not toxigenic, consumer safety was ensured (Scheuplein et al., 2007). PG catalyzes the deamidation of proteins at glutamine residues in both short peptide chains and proteins without causing side reactions like other deamidation enzymes, but it does not deamidate asparaginyl residues or free glutamines (Yamaguchi and Yokoe, 2000). Deamidation of PG has been studied on a variety of food proteins, including soy protein isolate (Suppavorasatit et al., 2011; Jiang et al., 2022), coconut protein (Kunarayakul et al., 2018), wheat gluten (Yong et al., 2006), α-zein (Yong et al., 2004), glutenin and gliadin (Chen et al., 2019), Oat protein (Jiang et al., 2015), and evening primrose protein (Hadidi et al., 2021), resulting in improved protein solubility, emulsifying and foaming properties. Food products may lose flavor due to deamidation. Temthawee et al. (2020) reported that the lower binding affinifor vanillin flavor deterioration in high protein aqueous foods. In addition, the effect of deamidation on gluten antigenicity was investigated. The sequence Gln-Gln-Gln-Pro-Pro is thought to be the most likely candidate for the IgE-binding epitope in gluten, and gluten lost its antigenicity in proportion to the degree of deamidation (Kanerva et al., 2011).

Previous research concentrated on the application of PG rather than enzyme-producing strains. The only commercially available PG was from *Chryseobacterium proteolyticum* isolated from soil (Amano Enzyme Inc., Japan). Several researchers have developed the heterologous expression system for PG production. *E. coli*, *Bacillus subtilis*, *Bacillus licheniformis*, and *C. glutamicum* have been used for PG over-expression (Zhang et al., 2021). Although Ouyang et al. (2021) optimized PG expression in various *B. subtilis* strains, the maximum PG activity was only 1.10 U/ml. Lu et al. (2020) developed an *E. coli* expression system for the mass production of active PG (15 mg/L). In *B. licheniformis* CBBD302, GlmU-R-mediated PG expression in active form with a maximum yield of 6.8 U/ml in a 25-L bioreactor (Niu et al., 2019). Yin et al. (2021) developed a combinatorial engineering method for high-yield production of PG in *B. subtilis*. PG activity reached 3.23 and 7.07 U/mL in shaken-flask cultures and fed-batch fermentation, respectively. However, in terms of safety, yield, and cost, the heterologous expression system fell short of meeting the needs of the food industry. As a result, screening high PG-producing strains from nature is critical.

Qu et al. (2018) isolated a PG-producing strain *C. proteolyticum* QDH1265 from soils described the entire genome sequence and the genomic information. Its enzyme activity was approximately 0.34 ± 0.01 U/mL. However, studies on wild PG-producing strains have been few and under-reported until now. PG has a wide range of applications in the food industry. However, PG currently has few industrial applications because of its low production by *C. proteolyticum*. As a result, improving its production with wild-type strains is critical for large-scale industrial applications. The present study isolated a new PG-producing strain *C. cucumeris* ZYF120413-7 from rice field soil in China. The purification and amino acid sequence of PG, enzymatic properties and deamidation on soy protein isolate (SPI) were investigated. The strain ZYF120413-7 expanded the wild-type strains resource for PG-producing strains, and the enhanced solubility of deamidated SPI demonstrated that PG from ZYF120413-7 can be efficiently used for proteins deamidation to improve their functional properties in the food industry.

## Materials and methods

### Bacterial strains and chemicals

The *Chryseobacterium cucumeris* ZYF120413-7 strain was isolated from rice field soil according to the method of Yamaguchi and Yokoe (2000) and stored in the Food and Microbial Technology Laboratory, School of Life Sciences, East China Normal University. SP-Sepharose FastFlow, Sephacryl S-100 HR pre-packed columns and the hollow fiber cross-flow filtration cartridges (UFP-5-C-3MA, MW 5KD) were purchased from GE Healthcare Life Sciences, United States. A Low molecular weight sodium dodecyl sulfate polyacrylamide gel electrophoresis (SDS-PAGE) marker was purchased from TaKaRa Bio Group, Japan. BCA assay kit was purchased from Beyotime Biotechnology, China. Polypeptone was purchased from Wako Pure Chemical Industries, Japan. SPI was obtained from Jinjing Biotechnology, China. Casein was obtained from Mercury Trading (Shanghai) Co., Ltd. Other analytical grade reagents were purchased from Sinopharm Group Chemical Reagent Co. Ltd. (Shanghai, China).

### Culture media and growth conditions

The screening medium was composed of (g/L) 1.0 carboxybenzoxy (Cbz)-Gln-Gly, 5.0 glucose, 0.2 KH_2_PO_4_, 0.2 MgSO_4_·7H_2_O, 0.1 NaCl, 0.02 CaCl_2_, 0.002 FeSO_4_·7H_2_O, 0.005 NaMO_4_· 2H_2_O, 0.005% NaWO_4_·4H_2_O, 0.005% MnSO_4_·4H_2_O, and 0.1 CuSO_4_·5H_2_O, and the pH was adjusted to 8.0 with 1 M NaOH. The seed culture medium was composed of (g/L) 10.0 polypeptone, 2.0 yeast-extract, 1.0 MgSO_4_·7H_2_O, and the pH was adjusted to 7.0 with 1 M NaOH. The fermentation medium was composed of (g/L) 5.0 lactose, 10.0 polypeptone, 1.7 Na_2_HPO_4_·H_2_O, 0.25 KH_2_PO_4_, 0.25 MgSO_4_·7H_2_O, and 0.05 FeSO_4_·7H_2_O, and the pH was adjusted to 7.2 (Yamaguchi and Yokoe, 2000). The slant culture of *C. cucumeris* ZYF120413-7 was transferred into a 50 ml seed medium in a 250 ml Erlenmeyer flask. After 12 h of culture at 30°C and 200 rpm, 2 ml of the cultures were inoculated into 48 ml of the fermentation medium in a 250 ml Erlenmeyer flask. The cultures were incubated in the constant temperature incubator shaker for 15 h at 30°C and 200 rpm (Zhichu, Shanghai, China), and the PG was in the culture broth.

### Morphology and physiology identification of ZYF120413-7

The strain was growing in LB agar medium at 30°C for 16 h, and the Gram staining and spore staining were tested according to standard procedures (Cappuccino and Sherman, 2004). Cell motility was tested in a semi-solid agar medium (Hopebio, Qingdao, China) by stab culture at 30°C for 48 h (Wang et al., 2016). The strain was cultured in 250-ml Erlenmeyer flasks containing a 50 ml seed culture medium at 30°C in the constant temperature incubator shaker (Zhichu, Shanghai, China) at 200 rpm for 12 h. The SEM analysis was performed using a scanning electron micrograph (EVO, Zeiss, Germany at 5.0 kV) according to method of Gao et al., 2020.

### PG activity and protein concentration determination

The indophenol method was used to determine the concentration of free ammonia after PG hydrolyzed aminoacyl groups on glutamine residues in proteins (Ouyang et al., 2021; Yin et al., 2021). The PG activity was determined using Cbz-Gln-Gly or casein as a substrate, and the detection procedures were according to the method of Ouyang et al. (2021). One unit of the enzyme was defined as the amount that released 1 μmol of ammonia per min under the reaction conditions. The protein concentration was determined with the BCA assay using bovine serum albumin as a standard (Yamaguchi and Yokoe, 2000).

### Partial purification of PG

The culture broth of *C. cucumeris* ZYF120413-7 was centrifuged at 12,000 *g* for 20 min at 4°C. The clear supernatant that contains PG was concentrated using an UFP-5-C-3MA hollow fiber ultrafiltration system (GE Healthcare Life Science, United States). Four volumes of cold ethanol were added to the concentrated solution and it was kept at 4°C for 2 h. The precipitate was collected by centrifugation at 12,000 *g* for 10 min and then resuspended in 20 mM sodium phosphate buffer (pH 6.5). The solution was dialyzed against the same buffer overnight, and filtrated through a 0.22 μ m pore size membrane to obtain a partially purified enzyme solution.

### Cation exchange chromatography and gel filtration chromatography

AKTA purifier, a protein purification system from GE Healthcare Life Science, United States, was used for purification studies. Cation exchange chromatography was performed using SP-Sepharose FastFlow 16/10 column. The partially purified solution was loaded onto the columns in start buffer (20 mM sodium phosphate buffer, pH 6.5), and then eluted by a linear NaCl gradient from 0 to 0.2 M at 1 ml/min. The PG activity in the eluted fractions was measured. PG-containing fraction was freeze-dried and resuspended with 2 ml of 20 mM sodium phosphate buffer (pH 6.5) containing 300 mM NaCl, and then filtrated with a 0.22 μm pore size membrane. The solution was applied to a gel filtration chromatography on a Sephacryl S-100 HR Hiprep 26/60 column. The column was equilibrated and eluted with 20 mM sodium phosphate buffer (pH 6.5) containing 300 mM NaCl at 1.3 ml/min. HiPrep26/10 desalting was used to desalt fractions containing PG activity, which were then freeze-dried. The PG powder was stored at −20°C for future research.

### SDS-PAGE analysis

Sodium dodecyl sulfate polyacrylamide gel electrophoresis was carried out at a constant voltage of 100 V using 12% separating gel and 5% stacking gel. Appropriately diluted 30 μl marker and samples were added into the wells. Molecular weight marker from TaKaRa Bio Group contained rabbit phosphorylase B (97.2 kDa), bovine serum albumin (66.4 kDa), ovalbumin (44.3 kDa), bovine carbonic anhydrase (29.0 kDa), trypsin inhibitor (20.1 kDa), and lysozyme (14.3 kDa). Gels were stained with 0.05% Coomassie Brilliant Blue R-250. Destaining was carried out with a solution of isopropanol and acetic acid.

### N-terminal amino acids sequence analysis

The purified enzyme on SDS-PAGE was electrotransferred onto a poly-vinylidence fluoride membrane (PVDF, Millipore) using a Bio-Rad Transblot apparatus at 50 V for 2 h with 3-(cyclohexylamino)-1-propanesulfonic acid (CAPS) buffer having a pH of 11.0. Protein spots were visualized by staining the membranes with 0.1% Coomassie Brilliant Blue R-250. Stained spots were excised and subjected to N-terminal sequence analysis on a model 492 protein sequencer (Applied Biosystems, United States; Fandi et al., 2001).

### Amplification and sequencing of PG gene

The PG gene of *C. cucumeris* ZYF120413-7 was amplified by PCR based on the sequence of PG gene in 9670^T^ found in NCBI. The forward primer WF1 (TGTCCATTCCGAAGGAATC) and the reverse primer WR2 (AAATCGGCGGTGCAGTCATTCATC) were used for the amplification. The PCR reactions systems were composed of 2 μl WF1 (10 nmol/L), 2 μl WR2 (10 nmol/L), 2 μl template DNA (145.2 ng/μl), 19 μl prime STAR premix (Takara), and 25 μl sterile ddH2O. The PCR procedure was carried out through 5 min of pre-denaturation at 95°C, with 35 cycles of denaturation at 95°C for 20 s, annealing at 55°C for 20 s, extension at 72°C for 30 s, followed by a final extension at 72°C for 10 min. The PCR products were purified by DNA Purification Kit (Tiangen, China) and sequenced by Sangon Biotech Technology Co., Ltd. (China).

### Effect of pH and temperature on PG activity and stability

The effect of pH on PG activity was determined by incubating the PG at 37°C for 20 min at different pH values (3 ~ 12) using Cbz-Gln-Gly as a substrate. The following buffers were used, 50 mM sodium acetate buffer (pH 3–6), 50 mM phosphate buffer (pH 7–9), and 50 mM Tris–HCl buffer (pH 10–12). The pH stability of the enzyme was determined by preincubating the PG solution at the various pH buffers mentioned above without substrate at 25°C for 24 h. The relative activities are expressed as a percentage of the maximum enzyme activity. The effect of temperature on PG activity was determined by incubating the reaction mixture of enzyme substrate for 20 min at different temperatures (20–80°C) at pH 6.5. PG thermostability was examined by preincubating PG solution without substrate for different time intervals (10, 30, and 60 min) at various temperatures (20, 30, 40, 50, 60, 70, and 80°C). After the termination of the reaction by cooling at 25°C, the substrate was added, the assay was performed and the relative activities were expressed as a percentage of the maximum enzyme activity.

### Effect of metal ions on PG activity

The effect of different metal ions or inhibitors on PG activity was determined by preincubating the PG at 37°C for 2 h with different metal ions (Fe^3+^, Mn^2+^, Mg^2+^, Ca^2+^, Zn^2+^, Na^+^, K^+^, and Li^+^) at three concentrations (1, 5, and 10 mM). The enzyme activity of the control group without adding metal ions was defined as 100%, and residual PG activity is expressed as a percentage of the control activity.

### Substrate specificities and kinetic parameters

The relative substrate specificity of PG toward proteins was determined under standard assay conditions. For kinetic studies, the initial rate of deamidation of Cbz-Gln-Gly at different substrate concentrations (0.625, 1.25, 2.5, 5, and 10 mM) was measured under standard assay conditions. According to Michaelis–Menten kinetics, plots of Lineweaver–Burk were made, and the kinetic constants Michaelis constant (*K_m_*) and maximum velocity (*V_max_*) were calculated by linear regression analyses (Xie et al., 2007).

### Deamidation of SPI

Deamidation of SPI was carried out in 50 mM phosphate buffer (pH 7.0) containing 10 mg/ml SPI samples and 30 U/g PG protein. The enzymatic reaction was carried over a period of time (0–18 h) at 37°C. The enzymes were deactivated by raising the temperature to 80°C for 10 min. The deamidated samples were gel electrophoresis analyzed by SDS-PAGE. The degree of deamidation (DD) and degree of hydrolysis (DH) of SPI were measured following the method of Suppavorasatit et al. (2011).

### Statistical analyses

Unless otherwise specified, all measurements were performed in triplicate. The results are presented as means ± SD. Statistical analysis was performed using GraphPad Prism 8 (GraphPad Software, Dan Diego, California, United States). Significance was considered at *p* < 0.05.

## Results

### Isolation of PG-producing strains

The protein-deamidating enzyme-producing strains were isolated from soils with the screening medium using Cbz-Gln-Gly as the sole nitrogen source. 168 bacteria were isolated from 726 soil samples and examined for PG productivity in culture supernatants with the fermentation medium. The strain ZYF120413-7, which originated from rice field soil in Mianyang, Sichuan Province, China, demonstrated the highest PG productivity and was used in the subsequent studies.

### Morphology and identification of the isolates

**Figure 1:**
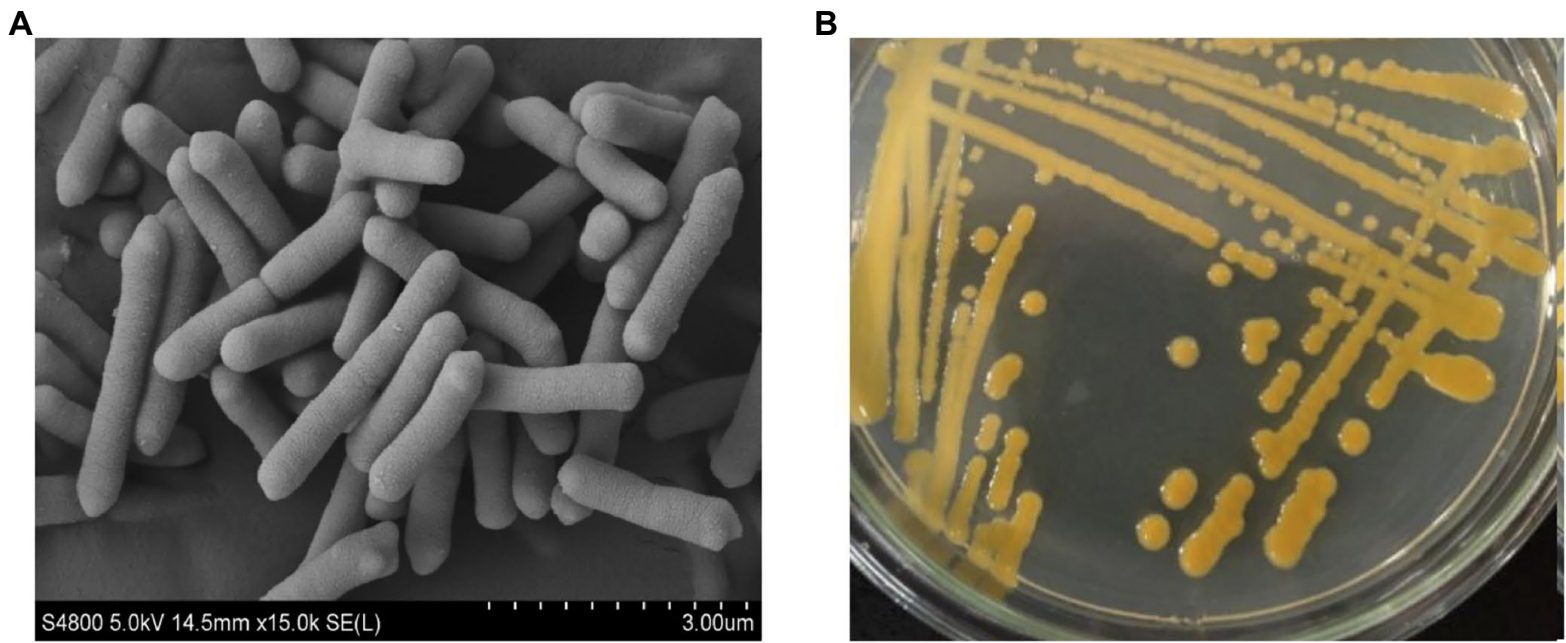
Scanning electron micrograph scanned at 15,000 magnification **(A)** and morphological characterization grown on seed culture medium agar at 30°C for 48 h **(B)** of strain ZYF120413-7.

The isolated strain ZYF120413-7 was Gram-negative, non-spore-forming and non-motile. The cells were rod-shaped, measuring 0.4 to 0.5 × 1.5 to 3.2 μm (Figure 1A) and occurred singly, in pairs, or in chains. The colonies of the strain ZYF120413-7 cultured on seed culture medium agar were golden, smooth, moist, butyrous, and approximately 2–4 mm in diameter (Figure 1B).

**Figure 2:**
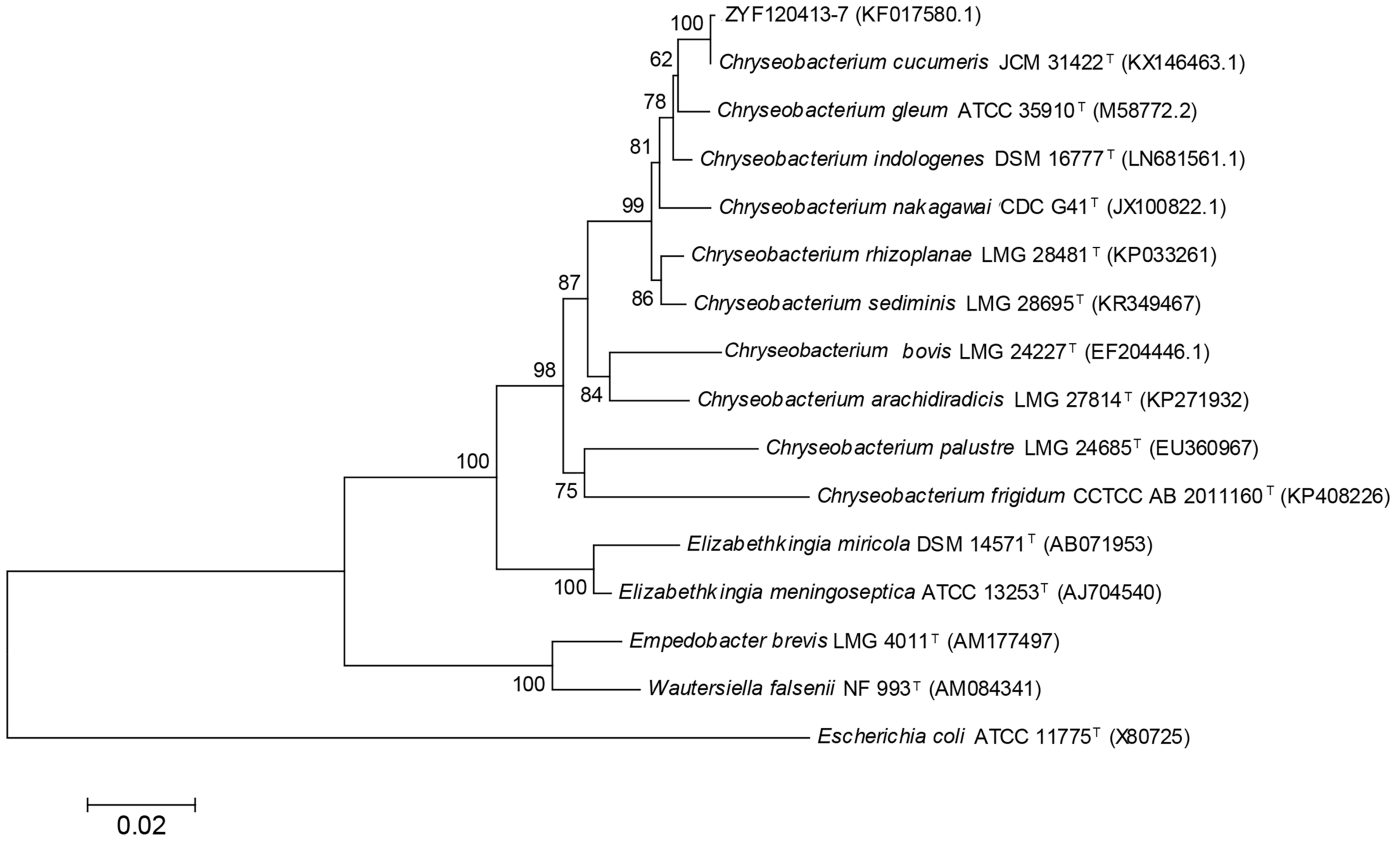
Phylogenetic tree based on 16S rRNA sequences of stain *Chryseobacterium cucumeris* ZYF120413-7. Phylogenetic relationships were inferred using the Neighbor-Joining method with 1,000 bootstrap replicates. Bootstrap values above 50% are shown next to the branches.

The 16S rRNA gene sequence was obtained for strain ZYF120413-7 (1,413 bp; GenBank accession number KF017580.1), and a phylogenetic tree was constructed (Figure 2). Phylogenetic analysis revealed that strain ZYF120413-7 was closely related to *C. cucumeris* and shared 99.93% identity based on a 1,000 bootstrap value. As a result, strain ZYF120413-7 was identified as *C. cucumeris* and given the name *C. cucumeris* ZYF120413-7.

### Batch culture profile of *Chryseobacterium cucumeris* ZYF120413-7

**Figure 3:**
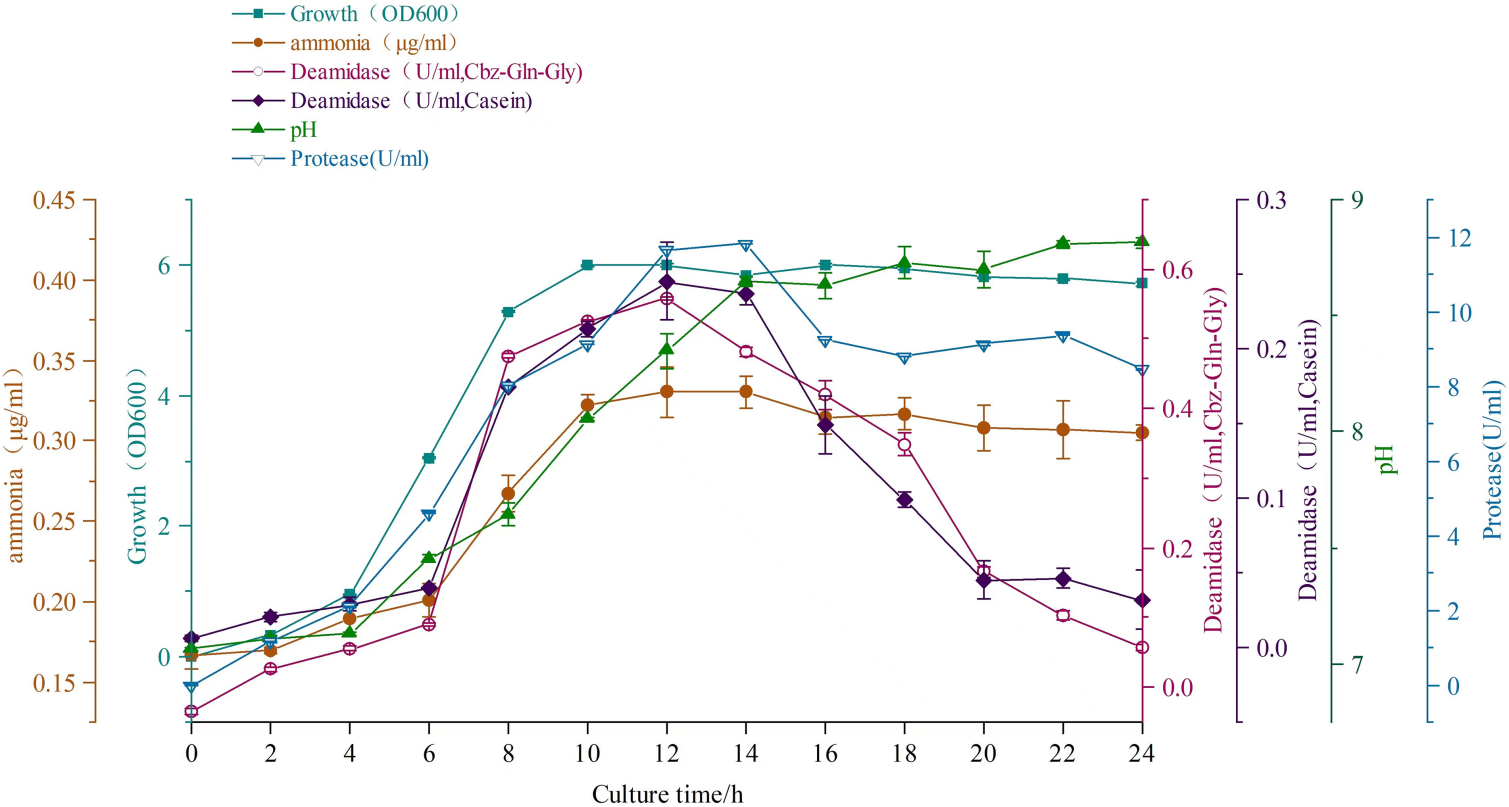
The batch culture profile of strain *Chryseobacterium cucumeris* ZYF120413-7.

The batch culture profile of strain *C. cucumeris* ZYF120413-7 is shown in Figure 3. The strain ZYF120413-7 grew rapidly from the start of culture, and the OD_600_ reached a maximum of 6.0 after 10 h and remained constant. Deamidating activities increased rapidly from 6 to 12 h, and then decreased rapidly. The maximum enzyme activities were observed at 12 h of culture with 0.557 U/ml on Cbz-Gln-Gly and 0.245 U/ml on casein. Protease activity was also detected, along with deamidating activities. Ammonia concentration increased from 4 to 12 h, reaching a maximum of 0.33 μg/ml. The pH increased from 6 h to a maximum of 8.6 at 14 h because of the increase in ammonia concentration.

### Purification of PG obtained from *Chryseobacterium cucumeris* ZYF120413-7

**Figure 4:**
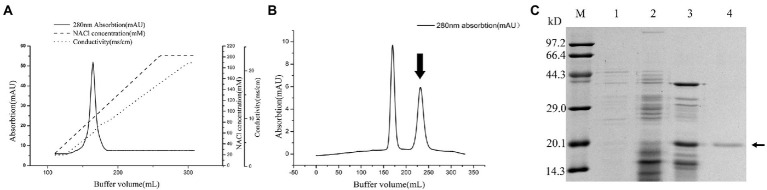
Chromatography of the crude Protein-glutaminase (PG) from *Chryseobacterium cucumeris* ZYF120413-7 subjected to cation exchange chromatography on SP Sepharose Fast Flow 16/10 column **(A)**, and gel filtration chromatography on Sephacryl S-100 HR 26/60 column **(B)**, the arrow indicated the active fraction containing PG activity, and sodium dodecyl sulfate polyacrylamide gel electrophoresis (SDS-PAGE) analysis of the PG purification steps **(C)**, M, molecular mass marker; 1, broth supernatant; 2, crude PG after ethanol precipitation; 3, crude PG after SP Sepharose FastFlow; 4, purified PG after Sephacryl S-100 HR, the arrow marks the position of PG.

The purification process of PG from the culture supernatant of *C. cucumeris* ZYF120413-7 is summarized in Table 1. The culture supernatant was concentrated 2.57-fold using a 5KD hollow fiber ultrafiltration membrane before ethanol precipitation. After ethanol precipitation, the concentrated supernatant yielded 46.16 mg of protein. The partially purified PG was subjected to SP Sepharose FastFlow cation exchange chromatography, with one peak obtained in and the elution profiles for PG, as shown in Figure 4A, the PG activity of the fraction was 8.403 U/mg, with a recovery of 48.02%. The collected fraction was further purified by Sephacryl S-100 gel filtration chromatography (Figure 4B), and the second peak containing the highest PG (27.37 U/mg) was collected. The overall increase in specific activity was of 161.95-fold, with a recovery rate of 32.95% (Table 1). SDS-PAGE revealed that the purified PG was a monomeric protein with an MW of 20 kD (Figure 4C).

**Table 1:** Summary of purification of the PG from *Chryseobacterium cucumeris* ZYF120413-7.

Steps	Total protein (mg)	Total activity (U)	Specific activity (U/mg)	Purification (fold)	Recovery (%)
Culture supernatant	968.25	163.63	0.169	1*	100**
Ultrafiltration	322.83	140.43	0.435	2.57	85.82
Ethanol	46.16	104.74	2.269	13.43	64.01
SP Sepharose	9.35	78.57	8.403	49.72	48.02
Sephacryl S-100	1.97	53.92	27.37	161.95	32.95

* The purification ratio of culture supernatant was defined as 1.00.

** The enzyme activity recovery was 100%.

### Identification of PG obtained from *Chryseobacterium cucumeris* ZYF120413-7

**Figure 5:**
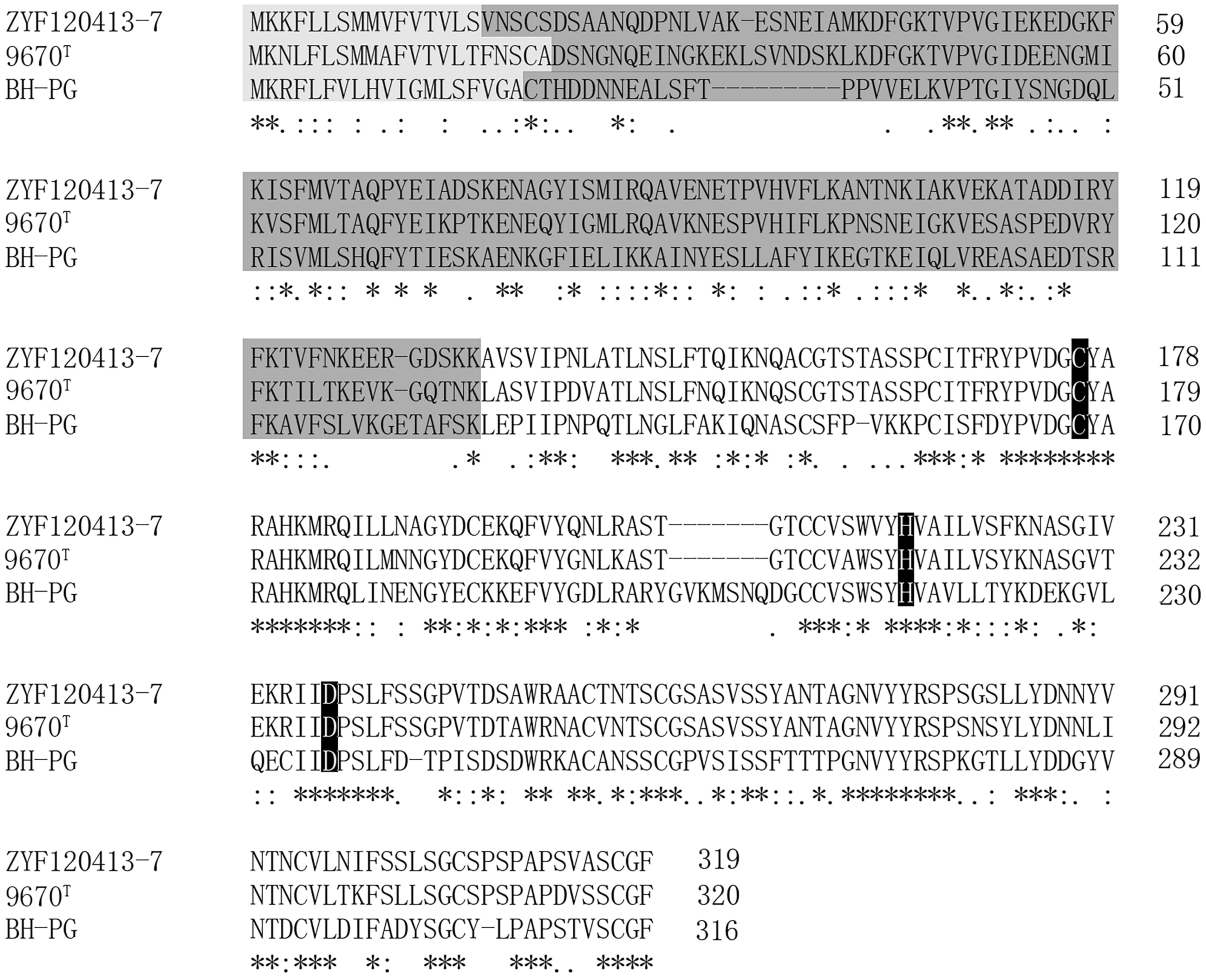
Alignment of the PG from *Bacteroides helcogenes* (BH-PG; UniProt ID: E6SWX4), *Chryseobacterium proteolyticum* (9670^T^, UniProt ID: Q9AQQ8), and *Chryseobacterium cucumeris* ZYF120413-7 (ZYF120413-7; GenBank ID: ON408292). The symbols denoting the degree of conservation are as follows: (“*”) identical residues; (“:”) conserved substitutions; (“.”) semi-conserved substitutions. Light gray indicates the predicted signal peptide, dark gray indicates the predicted pro-region, and others indicate the mature sequences. Amino acids indicated in white on the black background are the predicted amino acids of the active site.

The PG gene in *C. cucumeris* ZYF120413-7 was 960 bp, and could be translated to 319 amino acids. The N-terminal amino acid sequence of the first eight residues was determined as AVSVIPNL. The mature PG began with the 135th amino acid in the deduced amino acid sequence of the open reading frame, implying that the first 134 amino acids comprise a pre-pro-sequence. The first 16 amino acids of the putative pre-pro-sequence exhibited characteristics of a typical signal peptide, whereas the signal peptide of PG from *C. cucumeris* ZYF120413-7 contained 21 amino acids. The PG from *C. cucumeris* ZYF120413-7 was predicted to be synthesized as a pre-pro-protein containing a putative signal peptide of 16 amino acids, a pro-PG of 118 amino acids, and a mature PG of 185 amino acids. Figure 5 shows the alignment of the amino acids of PG from strain ZYF120413-7, 9670^T^, and BH-PG. The amino acid sequence identity of PG from *C. cucumeris* ZYF120413-7 to *C. proteolyticum* 9670^T^ and BH-PG was 74 and 45%, respectively.

### Effect of pH and temperature on PG activity

**Figure 6:**
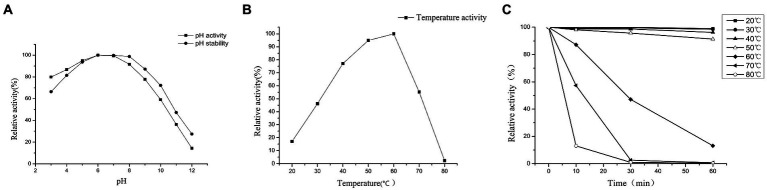
pH profile and pH stability **(A)**, temperature profile **(B)**, and thermostability **(C)** of PG.

Maximum PG activity was observed between pH 5 and 8, with an optimum at pH 6 (Figure 6A). The pH stability of PG was determined in a pH range of 3–12 at 25°C for 24 h incubation period (Figure 6A). PG remained stable over a wide pH range of 3–12. The residual activity at pH 3 was approximately 66.42% and remained above 98.82% up to pH 8, but increasing the pH beyond 9 resulted in a rapid decrease in enzyme activity, approximately 47.27% at pH 11 and 27.43% at pH 12. The effect of temperature on the enzyme substrate Cbz-Gln-Gly reaction was investigated. The PG activity increased with the increasing reaction temperature until the maximum activity was reached at 60°C. When the temperature exceeded 60°C, the PG activity rapidly decreased rapidly, and after 20 min of pre-incubation, inactivity was observed at 80°C (Figure 6B). The PG exhibited complete heat stability for 60 min at 40 and 50°C, but the enzyme lost its activity rapidly at 60, 70, and 80°C. After 30 min at 70 and 80°C, the PG activity was reduced by 97.5 and 99.1%, respectively (Figure 6C).

### Effect of metal ions on PG activity

Table 2 shows the effect of different metal ions on PG activity. Among the metal ions tested, Na^2+^ had a little activating effect on PG activity. Ca^2+^ had no effect on PG activity, and Fe^3+^, Mn^2+^, Mg^2+^, Ca^2+^, Zn^2+^, K^+^, and Li^+^ had slight inhibitory effects on PG activity, less than 5% when compared to the control.

**Table 2:** Effects of different metal ions on the PG activity from *C. cucumeris* ZYF120413-7.

Metal ions	Relative activity (%, Mean ± SD, CV)		
	Concentration (mM)		
	1	5	10
control	100	100	100
Fe^3+^	94.08 ± 0.26, 0.28	94.71 ± 0.25, 0.26	93.67 ± 1.18, 1.26
Mn^2+^	94.73 ± 0.26, 0.27	92.64 ± 0.35, 0.38	92.84 ± 0.14, 1.51
Mg^2+^	97.73 ± 4.14, 4.24	96.02 ± 0.55, 0.57	96.11 ± 1.73, 1.80
Ca^2+^	99.52 ± 1.50, 1.51	100.89 ± 0.67, 0.66	100.17 ± 1.09, 1.09
Zn^2+^	99.46 ± 1.07, 1.08	97.33 ± 0.87, 0.89	98.29 ± 1.20, 1.22
Na^+^	103.11 ± 0.51, 0.49	106.24 ± 0.33, 0.31	104.48 ± 1.27, 1.22
K^+^	97.23 ± 0.75, 0.77	96.30 ± 1.24, 1.29	95.17 ± 1.04, 1.09
Li^+^	93.70 ± 0.45, 0.48	94.59 ± 2.44, 2.58	93.40 ± 1.00, 1.07

### Substrate specificity and kinetic parameter

**Figure 7:**
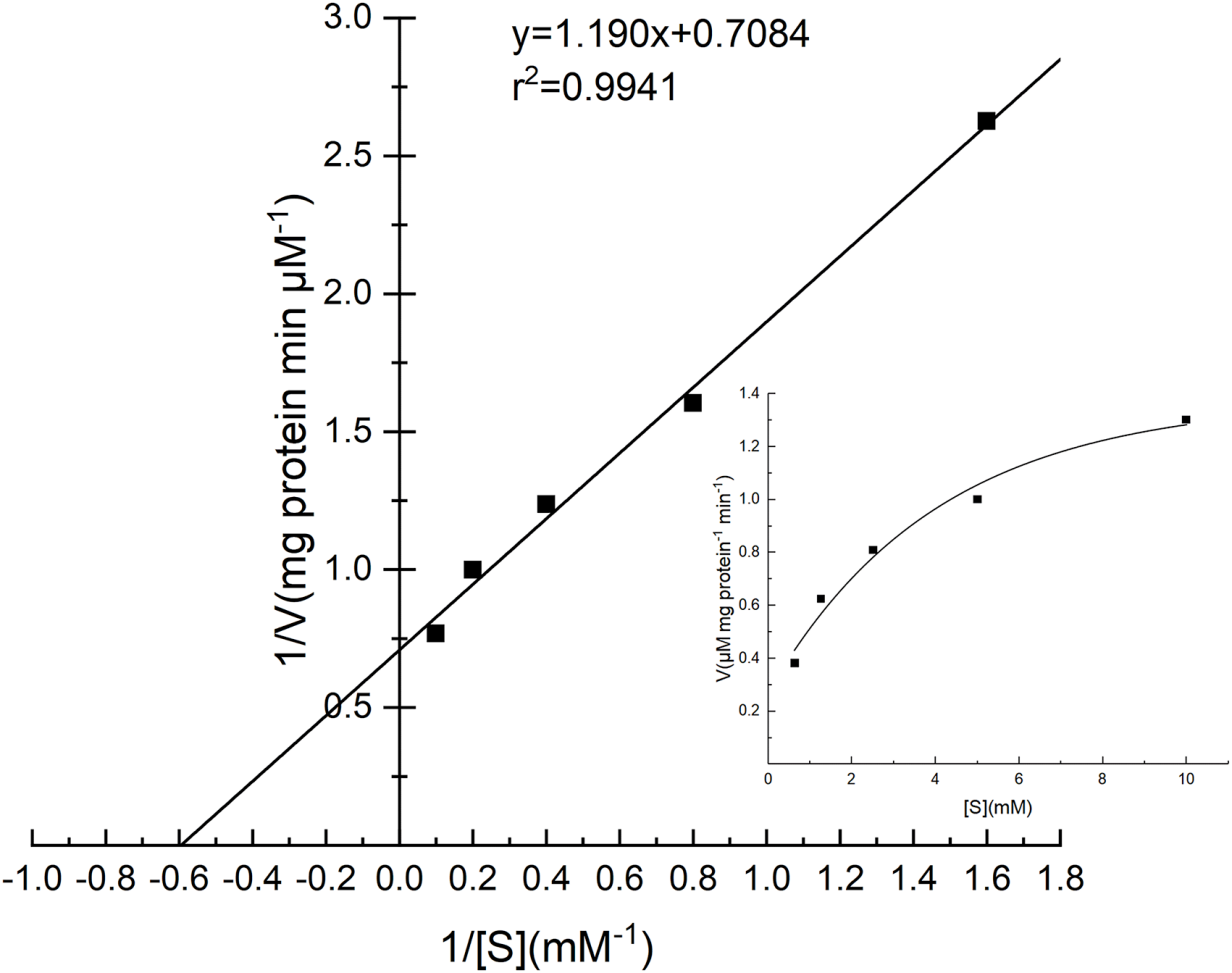
Lineweaver–Burk plot for the determination of *K_m_* and *V_max_* for PG from *Chryseobacterium cucumeris* ZYF120413-7.

The specific activities of PG toward four substrates were investigated, and the results are shown in Table 3. Cbz-Gln-Gly was the best substrate, followed by soy protein isolate, while gelatin and bovine serum albumin performed poorly. The Lineweaver–Burk plot of PG is shown in Figure 7, and the *K_m_* and *V_max_* values of PG calculated from Lineweaver–Burk plot were 1.68 mM and 1.41 μM mg protein^−1^ min^−1^, respectively.

**Table 3:** The PG specificity toward different substrates.

Substrate	Specific activity (U/mg)	Coefficient of variation (%)
Cbz-Gln-Gly	1.98 ± 0.06	3.03
Gelatin	0.12 ± 0.01	8.33
Soy protein isolate	0.42 ± 0.01	2.38
Bovine serum albumin	0.03 ± 0.01	33.33

### DD and DH of SPI by PG

**Figure 8:**
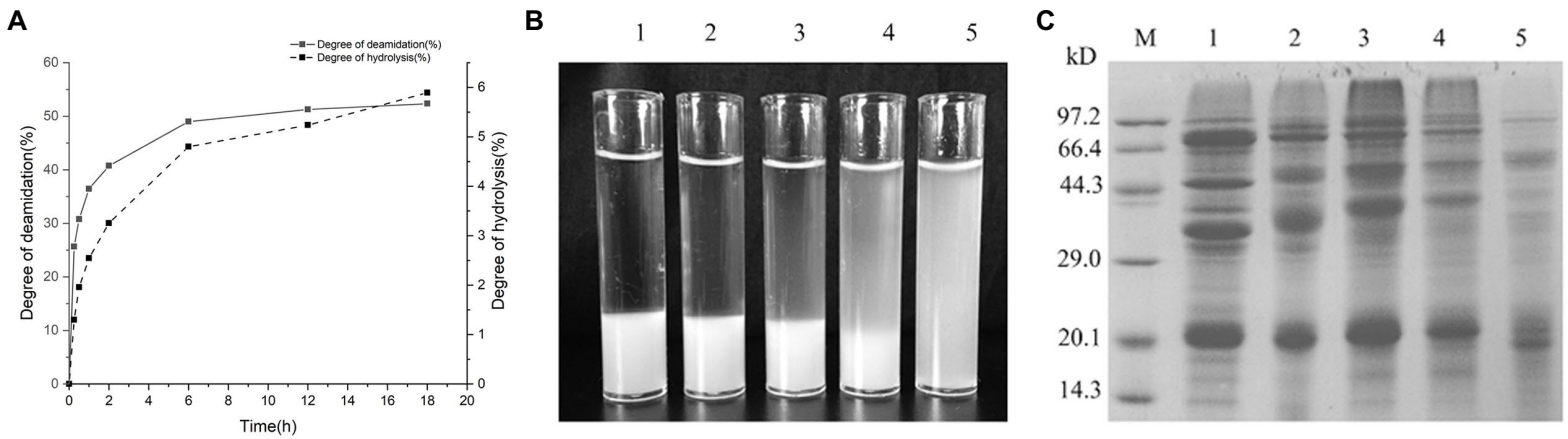
Changes in DD and DH of soy protein isolate (SPI) as a function of reaction time **(A)**; appearance of 1% deamidated SPI with PG for 0, 0.5, 2, 6, and 18 h in phosphate buffer (200 mM, pH 7.0), respectively **(B)**, and SDS-PAGE of SPI deamidated with PG for 0, 0.5, 2, 6, and 18 h, respectively **(C)**.

Soy protein isolate is widely used in the food industry due to its nutritional and functional properties. Enzymatic deamidation is a promising method for improving the functional properties of SPI, such as solubility, emulsibility, and others. SPI was deamidated by purified PG, and changes in the DD and DH of SPI as a function of reaction time are shown in Figure 8A. DD rapidly increased to 40.75% within the first 2 h, then gradually increased to 52.35% after 18 h. DH was lower than DD at all time points, but it showed similar pattern. DH increased rapidly within the first 2 h to approximately 3.25%, then gradually increased to 5.89% at 18 h. Figure 8B shows the appearance of deamidated SPI dispersions. SPI deamidated for 2 h or less showed clear solid–liquid separation. The samples deamidated for 6 h began to show a mixture of turbid solution and precipitate. For 18 h of deamidation, a homogeneous turbid solution was obtained. SDS-PAGE profiles of SPI deamidated for 0 h (control), 0.5, 2, 6, and 18 h are shown in Figure 8C. The gel pattern clearly showed that the deamidated SPI (lanes 1–5) differed from the control SPI. Deamidated SPI band patterns revealed an upward shift to higher molecular mass units.

## Discussion

In this study, PG was produced from *C. cucumeris* isolated from rice field soil in China. The maximum PG activity of *C. cucumeris* ZYF120413-7 was 0.557 U/ml on Cbz-Gln-Gly and 0.245 U/ml on casein after 12 h of culture. According to Yamaguchi and Yokoe (2000), the maximum PG activity of strain 9670^T^ was 0.258 U/ml on Cbz-Gln-Gly and 0.228 U/ml after 24 h of culture. Strain ZYF120413-7 was more suitable for PG production than strain 9670^T^ because of its higher PG activity and shorter culture time. The highest OD_600_ of strain 9670^T^ was around 2.1 after 20 h of culture. However, OD_600_ reached a maximum of 6.0 after only 10 h of culture for strain ZYF120413-7. The rapid growth may be the reason for the high PG yield of strain ZYF120413-7. Because there are few wild PG-producing strains, some genetically engineered strains have been developed. Zhang et al. (2021) reviewed the PG-producing strains that have been identified to date, including heterologous expressed PG. The heterologous expressed PG in *B. licheniformis* CBBD302 yielded 6.8 U/ml (Niu et al., 2019) and in *B. subtilis*, it yielded 7.07 U/ml by combined optimization of the promoter, signal peptide, and culture medium (Yin et al., 2021). However, the heterologous expression of PG could not meet the safety requirements of the food industry. As a result, screening high-yield wild PG-producing strains is critical.

Yamaguchi et al. (2001) purified PG from *C. proteolyticum* strain 9670^T^ through four steps, and PG was enriched 153-fold with a recovery of 30.3%. In this study, purified PG was concentrated 161.95-fold with a recovery of 32.95%. Ethanol precipitation is one of the most common methods for protein purification in the food industry, and it is an effective method to purify PG from the culture supernatant of *C. cucumeris* ZYF120413-7. This result provided an approach for PG purification on the industrial scale.

In order to elucidate the mechanism of PG-catalyzed deamidation specifically toward the substrate of protein-bound glutaminyl residues, the solution structure of PG was determined by nuclear magnetic resonance. The catalytic cysteine residue of PG was located at the deep negatively charged pocket in the shallow cleft which prefers the glutaminyl residue as a sole substrate, and the depth of the cleft fitted the length of a glutaminyl side chain but not the length of an asparaginyl side chain (Kumeta et al., 2010). The regions around the predicted amino acids (Cys, His, and Asp) of the active site are highly conserved (Figure 5; black; Horstmann et al., 2020), and PG seems to be evolutionally related to transglutaminase (Hashizume et al., 2011).

During the culture of strain ZYF120413-7, the pH value and ammonia concentration increased with PG production, and ammonia could be released from polypeptone in the medium by deamidating activities. Both PG activity and protease activity were produced synchronously during the culture strain ZYF120413-7, which agreed with the findings of Yamaguchi and Yokoe (2000). The presence of protease in the fermentation broth hampered its application in the food industry and should be removed during the purification process. Deamidation by commercial PG products caused slight protein degradation in SPI (Suppavorasatit et al., 2011), soymilk (Suppavorasatit et al., 2013), and coconut protein (Kunarayakul et al., 2018).

Horstmann et al. (2020) recently reported a PG (BH-PG) gene sequence from *Bacteroides helcogenes* that was heterologously expressed in *Escherichia coli*. The amino acid sequence identity of PG from strain ZYF120413-7 was 74 and 45% similar to those from 9670^T^ and BH-PG, respectively. The mature domain of all three enzymes contained 185 amino acids. The PG from *C. cucumeris* ZYF120413-7 contains a 16 amino acid putative signal peptide and 118 amino acids pro-region. However, PG from 9670^T^ and BH-PG has a putative signal peptide sequence of 21 and 19 amino acids, as well as a pro-sequence of 114 and 108 amino acids. The predicted amino acids (Cys, His, and Asp) of the active site (Hashizume et al., 2011) were highly conserved (Figure 5; black). This provides the basis for further mutation using a site-directed strategy to obtain high-yield strains.

The optimum reaction temperature was 60°C for PG from strain ZYF120413-7 and 9670^T^, and 50°C for BH-PG (Yamaguchi and Yokoe, 2000; Hashizume et al., 2011). In this study, the residual PG activity was 12% after 10 min of incubation at 80°C and the PG from strain 9670^T^ lost its activity completely under the same conditions (Yamaguchi and Yokoe, 2000). The optimum pH of PG from strain ZYF120413-7 was between pH 5 and 8, the optimum pH of PG for strain 9670^T^ was between 5 and 7 (Yamaguchi and Yokoe, 2000), and the highest activity of BH-PG was observed at pH 5.5 (Hashizume et al., 2011). As a result, the PG from strain ZYF120413-7 had higher thermostability and a wider optimum pH range, which were advantages for use in food processing.

Soy protein is regarded as an alternative to animal-derived protein due to its favorable functional and nutritional profiles. Suppavorasatit et al. (2011) reported that DD of SPI deamidated by PG was around 40% within the first 2 h and around 53% after 24 h, which was similar to the result of this study (Figure 8A). The deamidated SPI showed improved solubility in acidic and neutral conditions, as well as emulsification properties and foaming capacity (Suppavorasatit et al., 2011). Preheating treatment at 70°C could accelerate the PG deamidation reaction of SPI (Jiang et al., 2022). According to the current study, deamidated SPI showed shifting bands toward the upper part of the gel of SDS-PAGE analysis. This shifting phenomenon was observed in the other proteins deamidated by PG (Gu et al., 2001; Yong et al., 2004, 2006) due to the increase in the negative charge of deamidated proteins (Yong et al., 2004). Deamidation of SPI with improved functional properties can be used for various purposes in the food industry. For example, protein solubility was increased in the deamidation of soymilk at weakly acidic conditions (pH 5.0; Suppavorasatit et al., 2013), which could benefit the formulation of weakly acidic soymilk beverages.

## Conclusion

A novel PG-producing strain *C. cucumeris* ZYF120413-7 was obtained, with a high PG yield and a short culture time due to its rapid growth and increased biomass. The PG from strain ZYF120413-7 had higher thermostability and a wider pH range, making it suitable for deamidation of food proteins. The deamidated SPI improved solubility and has the potential to be used in protein-fortified beverages. The PG-producing strain *C. cucumeris* ZYF120413-7 expanded the wild PG-producing strains. Further research should be conducted to investigate the application and its impact on the functional properties of food. Mutagenesis breeding and high-throughput screening methods can be used to improve the PG enzyme activity of strain ZYF120413-7. Furthermore, optimizing the culture medium composition and fermentation conditions can be used to improve PG enzyme activity. PG can improve the functional properties of plant-based protein, which is becoming more popular due to its low cost and environmentally friendly production process.

## Data availability statement

The datasets presented in this study can be found in online repositories. The names of the repository/repositories and accession number(s) can be found at: https://www.ncbi.nlm.nih.gov/genbank/, ON408292.

## Author contributions

The work was conceived and designed by JH and HG. Experiments were done by RQ, TD, YZ, and LK. Data analysis and verification were done by TD, JW, AT, WO, and CJ. The manuscript was drafted by RQ, TD, JN, and ZL. The manuscript was revised by ZC, DJ, JH, and HG. All authors contributed to the article and approved the submitted version.

## Funding

This work was supported by the East China Normal University (ECNU) Public Platform for Innovation (011) and the Instruments Sharing Platform of School of Life Sciences, ECNU. This work was sponsored by “Chenguang Program” supported by Shanghai Education Development Foundation and Shanghai Municipal Education Commission (project number 19CGB13).

## Conflict of interest

The authors declare that the research was conducted in the absence of any commercial or financial relationships that could be construed as a potential conflict of interest.

## Publisher’s note

All claims expressed in this article are solely those of the authors and do not necessarily represent those of their affiliated organizations, or those of the publisher, the editors and the reviewers. Any product that may be evaluated in this article, or claim that may be made by its manufacturer, is not guaranteed or endorsed by the publisher.

## References

[ref1] Cappuccino J. Sherman N. (2004). Microbiology, Laboratory Manual. New Delhi, India: Person Education Publication.

[ref2] Chen X. Fu W. Y. Luo Y. C. Cui C. Suppavorasatit I. Liang L. (2021). Protein deamidation to produce processable ingredients and engineered colloids for emerging food applications. Compr. Rev. Food Sci. Food Saf. 20, 3788–3817. doi: 10.1111/1541-4337.12759, PMID: 34056849

[ref3] Chen B. Wang Y. R. Fan J. L. Yang Q. Chen H. Q. (2019). Effect of glutenin and gliadin modified by protein-glutaminase on retrogradation properties and digestibility of potato starch. Food Chem. 301:125226. doi: 10.1016/j.foodchem.2019.125226, PMID: 31357003

[ref4] Fandi K. G. Ghazali H. M. Yazid A. M. Raha A. R. (2001). Purification and N-terminal amino acid sequence of fructose-6-phosphate phosphoketolase from Bifidobacterium longum BB536. Lett. Appl. Microbiol. 32, 235–239. doi: 10.1046/j.1472-765X.2001.00895.x, PMID: 11298932

[ref5] Fang L. Y. Xiang H. Sun-Waterhouse D. Cui C. Lin J. J. (2020). Enhancing the usability of pea protein isolate in food applications through modifying its structural and sensory properties via Deamidation by Glutaminase. J. Agric. Food Chem. 68, 1691–1697. doi: 10.1021/acs.jafc.9b06046, PMID: 31951402

[ref6] Gao H. L. Xie F. R. Zhang W. Tian J. T. Zou C. J. Jia C. F.. (2020). Characterization and improvement of curdlan produced by a high-yield mutant of *Agrobacterium* sp. ATCC 31749 based on whole-genome analysis. Carbohydr. Polym. 245:116486. doi: 10.1016/j.carbpol.2020.116486, PMID: 32718606

[ref7] Gu Y. S. Matsumura Y. Yamaguchi S. Mori T. (2001). Action of protein-glutaminase on alpha-lactalbumin in the native and molten globule states. J. Agric. Food Chem. 49, 5999–6005. doi: 10.1021/jf010287z, PMID: 11743799

[ref8] Hadidi M. Ibarz A. Pouramin S. (2021). Optimization of extraction and deamidation of edible protein from evening primrose (*Oenothera biennis* L.) oil processing by-products and its effect on structural and techno-functional properties. Food Chem. 334:127613. doi: 10.1016/j.foodchem.2020.127613, PMID: 32711281

[ref9] Hamada J. S. (1991). Peptidoglutaminase deamidation of proteins and protein hydrolysates for improved food use1. J. Am. Oil Chem. Soc. 68, 459–462. doi: 10.1007/BF02663813

[ref10] Hashizume R. Maki Y. Mizutani K. Takahashi N. Matsubara H. Sugita A.. (2011). Crystal structures of protein Glutaminase and its pro forms converted into enzyme-substrate complex. J. Biol. Chem. 286, 38691–38702. doi: 10.1074/jbc.M111.255133, PMID: 21926168 PMC3207460

[ref11] Horstmann G. Ewert J. Stressler T. Fischer L. (2020). A novel protein glutaminase from *Bacteroides helcogenes*-characterization and comparison. Appl. Microbiol. Biotechnol. 104, 187–199. doi: 10.1007/s00253-019-10225-2, PMID: 31773205

[ref12] Jiang Z. Q. Sontag-Strohm T. Salovaara H. Sibakov J. Kanerva P. Loponen J. (2015). Oat protein solubility and emulsion properties improved by enzymatic deamidation. J. Cereal Sci. 64, 126–132. doi: 10.1016/j.jcs.2015.04.010

[ref13] Jiang Y. Q. Wang Z. J. He Z. Y. Zeng M. M. Qin F. Chen J. (2022). Effect of heat-induced aggregation of soy protein isolate on protein-glutaminase deamidation and the emulsifying properties of deamidated products. LWT. Food Sci. Technol. 154:112328. doi: 10.1016/j.lwt.2021.112328

[ref14] Kanerva P. Brinck O. Sontag-Strohm T. Salovaara H. Loponen J. (2011). Deamidation of gluten proteins and peptides decreases the antibody affinity in gluten analysis assays. J. Cereal Sci. 53, 335–339. doi: 10.1016/j.jcs.2011.02.003

[ref15] Kumeta H. Miwa N. Ogura K. Kai Y. Mizukoshi T. Shimba N.. (2010). The NMR structure of protein-glutaminase from Chryseobacterium proteolyticum. J. Biomol. NMR 46, 251–255. doi: 10.1007/s10858-010-9399-7, PMID: 20195702

[ref16] Kunarayakul S. Thaiphanit S. Anprung P. Suppavorasatit I. (2018). Optimization of coconut protein deamidation using protein-glutaminase and its effect on solubility, emulsification, and foaming properties of the proteins. Food Hydrocoll. 79, 197–207. doi: 10.1016/j.foodhyd.2017.12.031

[ref17] Lipan L. Rusu B. Sendra E. Hernández F. Vázquez-Araújo L. Vodnar D. C.. (2020). Spray drying and storage of probiotic-enriched almond milk: probiotic survival and physicochemical properties. J. Sci. Food Agric. 100, 3697–3708. doi: 10.1002/jsfa.10409, PMID: 32248520

[ref18] Liu X. Wang C. Zhang X. W. Zhang G. Q. Zhou J. W. Chen J. (2022). Application Prospect of protein-Glutaminase in the development of plant-based protein foods. Foods 11:11. doi: 10.3390/foods11030440 PMC8834458 35159590

[ref19] Lu X. Poon T. C. W. Zhang H. M. (2020). Mass production of active recombinantChryseobacterium proteolyticumprotein glutaminase in *Escherichia coli*using a sequential dual expression system and one-step purification. IUBMB Life 72, 2391–2399. doi: 10.1002/iub.2358, PMID: 32827356

[ref20] Niu D. D. Li C. Y. Wang P. Huang L. McHunu N. P. Singh S.. (2019). Twin-arginine signal peptide of *Bacillus licheniformis* Glm U efficiently mediated secretory expression of protein glutaminase. Electron. J. Biotechnol. 42, 49–55. doi: 10.1016/j.ejbt.2019.10.006

[ref21] Ohtsuka T. Umezawa Y. Nio N. Kubota K. (2001). Comparison of deamidation activity of transglutaminases. J. Food Sci. 66, 25–29. doi: 10.1111/j.1365-2621.2001.tb15576.x

[ref22] Ouyang X. Y. Liu Y. J. Qu R. D. Tian M. Yang T. Zhu R.. (2021). Optimizing protein-Glutaminase expression in *Bacillus subtilis*. Curr. Microbiol. 78, 1752–1762. doi: 10.1007/s00284-021-02404-0, PMID: 33740115

[ref23] Qu R. D. Zhu X. Y. Tian M. Liu Y. J. Yan W. J. Ye J.. (2018). Complete genome sequence and characterization of a protein-Glutaminase producing strain, Chryseobacterium proteolyticum QSH1265. Front. Microbiol. 9:1975. doi: 10.3389/fmicb.2018.01975, PMID: 30233508 PMC6132073

[ref24] Scheuplein R. J. Mizutani A. Yamaguchi S. (2007). Studies on the non-pathogenicity of Chryseobacterium proteolyticum and on the safety of the enzyme: protein-glutaminase. Regul. Toxicol. Pharmacol. 49, 79–89. doi: 10.1016/j.yrtph.2007.06.001, PMID: 17630060

[ref25] Schreuders F. K. G. Dekkers B. L. Bodnar I. Erni P. Boom R. M. van der Goot A. J. (2019). Comparing structuring potential of pea and soy protein with gluten for meat analogue preparation. J. Food Eng. 261, 32–39. doi: 10.1016/j.jfoodeng.2019.04.022

[ref26] Suppavorasatit I. De Mejia E. G. Cadwallader K. R. (2011). Optimization of the enzymatic Deamidation of soy protein by protein-Glutaminase and its effect on the functional properties of the protein. J. Agric. Food Chem. 59, 11621–11628. doi: 10.1021/jf2028973, PMID: 21954863

[ref27] Suppavorasatit I. Lee S. Y. Cadwallader K. R. (2013). Effect of enzymatic protein Deamidation on protein solubility and flavor binding properties of soymilk. J. Food Sci. 78, C1–C7. doi: 10.1111/j.1750-3841.2012.03012.x, PMID: 23277916

[ref28] Temthawee W. Panya A. Cadwallader K. R. Suppavorasatit I. (2020). Flavor binding property of coconut protein affected by protein-glutaminase: vanillin-coconut protein model. LWT. Food Sci. Technol. 130:109676. doi: 10.1016/j.lwt.2020.109676

[ref29] Wang Y. N. He W. H. An M. L. Tian W. Y. You X. Y. Ying F. Q.. (2016). Chryseobacterium zhengzhouense sp nov., isolated from groundwater of the well in a vegetable field, and emended description of the genus Chryseobacterium. Anton. Leeuw. Int. J. Gen. Mol. Microbiol. 109, 1299–1306. doi: 10.1007/s10482-016-0747-9 27522654

[ref30] Wilbanks R. (2017). Real vegan cheese and the artistic critique of biotechnology. Engag. Sci. Technol. Soc. 3, 180–205. doi: 10.17351/ests2017.53, PMID: 29951587 PMC6018000

[ref31] Xie J. J. Song K. K. Qiu L. He Q. Huang H. Chen Q. X. (2007). Inhibitory effects of substrate analogues on enzyme activity and substrate specificities of mushroom tyrosinase. Food Chem. 103, 1075–1079. doi: 10.1016/j.foodchem.2006.04.030

[ref32] Yamaguchi S. Jeenes D. J. Archer D. B. (2001). Protein-glutaminase from Chryseobacterium proteolyticum, an enzyme that deamidates glutaminyl residues in proteins - purification, characterization and gene cloning. Eur. J. Biochem. 268, 1410–1421. doi: 10.1046/j.1432-1327.2001.02019.x, PMID: 11231294

[ref33] Yamaguchi S. Yokoe M. (2000). A novel protein-deamidating enzyme from Chryseobacterium proteolyticum sp nov., a newly isolated bacterium from soil. Appl. Environ. Microbiol. 66, 3337–3343. doi: 10.1128/aem.66.8.3337-3343.2000, PMID: 10919788 PMC92152

[ref34] Yin X. X. Zhang G. Q. Zhou J. W. Li J. H. Du G. C. (2021). Combinatorial engineering for efficient production of protein-glutaminase in *Bacillus subtilis*. Enzym. Microb. Technol. 150:109863. doi: 10.1016/j.enzmictec.2021.109863, PMID: 34489022

[ref35] Yong Y. H. Yamaguchi S. Gu Y. S. Mori T. Matsumura Y. (2004). Effects of enzymatic deamidation by protein-glutaminase on structure and functional properties of alpha-zein. J. Agric. Food Chem. 52, 7094–7100. doi: 10.1021/jf040133u, PMID: 15537323

[ref36] Yong Y. H. Yamaguchi S. Matsumura Y. (2006). Effects of enzymatic deamidation by protein-glutaminase on structure and functional properties of wheat gluten. J. Agric. Food Chem. 54, 6034–6040. doi: 10.1021/jf060344u, PMID: 16881713

[ref37] Zhang G. Q. Ma S. J. Liu X. Yin X. X. Liu S. Zhou J. W.. (2021). Protein-glutaminase: research progress and prospect in food manufacturing. Food Biosci. 43:101314. doi: 10.1016/j.fbio.2021.101314

